# Colon‐Targeted Natural Polysaccharide‐Berberine Armored Hydrogel for the Treatment of Colitis

**DOI:** 10.1002/adhm.202404908

**Published:** 2025-06-25

**Authors:** Miao Guo, Bo Li, Hongyi Li, Yi Chen, Qin Yuan, Mingju Shui, Hefeng Zhou, Wei Hao, Shengpeng Wang

**Affiliations:** ^1^ State Key Laboratory of Quality Research in Chinese Medicine Institute of Chinese Medical Sciences University of Macau Macao 999078 China; ^2^ College of Pharmacy Chongqing College of Traditional Chinese Medicine Chongqing 402760 China; ^3^ Department of Bioengineering Zhuhai Campus of Zunyi Medical University Zhuhai 519090 China; ^4^ Science and Technology Innovation Center Guangzhou University of Chinese Medicine Guangzhou 510006 China

**Keywords:** berberine, gut microbiota, intestinal immune, Rhubarb polysaccharides, ulcerative colitis

## Abstract

The maintenance of gut immune homeostasis and microbial balance is pivotal in the pathogenesis and progression of ulcerative colitis (UC). Despite advances in therapy, effective UC management remains challenging due to the limited efficacy and significant side effects of conventional treatments. Inspired by the synergistic mechanisms of bioactive compounds in traditional Chinese medicine, a colon‐targeted hydrogel integrating rhubarb‐derived polysaccharides and berberine‐loaded dendrimers is engineered. This hydrogel self‐assembles via intermolecular hydrogen bonding and electrostatic interactions, enabling localized accumulation in colonic tissues to suppress aberrant immune activation and remodel the dysbiotic microbiome. Mechanistic studies reveal that the hydrogel potently promotes the polarization of anti‐inflammatory M2 macrophages while suppressing pro‐inflammatory cytokine secretion, resulting in significant amelioration of colitis symptoms in murine models. Importantly, the therapeutic intervention not only restored gut microbiota composition but also corrected metabolic disturbances, collectively contributing to the re‐establishment of intestinal homeostasis. The findings underscore the potential of this polysaccharide‐based hydrogel as an effective oral therapeutic strategy for UC while demonstrating the translational value of combining natural bioactive constituents for targeted drug delivery.

## Graphical abstract

In this research, a novel hydrogel system targeting the colon is developed, incorporating Rhubarb polysaccharides and berberine‐loaded dendrimer. This hydrogel, forms through intermolecular hydrogen bonding and electrostatic interactions, accumulates in colonic tissues, effectively alleviating pathological immune hyperactivation while modulating gut microbiota composition and associated metabolic profiles during ulcerative colitis (UC) treatment. The results underscore the potential clinical applications of this hydrogel system in UC management.

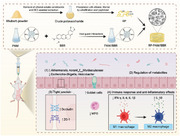

## 1 Introduction

The gut, the largest immune organ in the human body, may engage in excessive self‐protection against harmless antigens due to continuous exposure to the diet and the commensal microbiota, ultimately leading to chronic intestinal inflammation.^[^ ^1^ ^]^ Specifically, the recruitment and infiltration of immune cells, the secretion and accumulation of pro‐inflammatory cytokines,^[^ ^2^ ^]^ and dysbiosis of the gut microbiota^[^ ^3^ ^]^ can collectively lead to tissue damage and ulceration, thereby precipitating the onset of inflammatory bowel disease (IBD).^[^ ^4^ ^]^ IBD is broadly classified into ulcerative colitis (UC) and Crohn's disease (CD), distinguished by their distinct lesion patterns and anatomical distributions. UC, in particular, manifests with debilitating symptoms such as abdominal pain, bloody diarrhea, and systemic complications, severely compromising patients' quality of life and imposing substantial socioeconomic burdens.^[^ ^5^ ^]^

Currently, 5‐aminosalicylic acid (5‐ASA) and corticosteroids serve as first‐line clinical treatments. However, their non‐specific anti‐inflammatory properties can lead to various adverse effects.^[^ ^6^ ^]^ Furthermore, novel biological agents are often associated with high costs and substantial rates of non‐response and resistance.^[^ ^7^ ^]^ As research advances, combination therapy, rather than monotherapy, has shown significant and enduring efficacy for diseases characterized by complex pathogenic mechanisms and pathological processes.^[^ ^8^ ^]^ In particular, the “constituent synergy” that arises during the prescriptions of Chinese medicine presents notable potential for clinical application.^[^ ^9^ ^]^ Bioactive constituents derived from Chinese medicine can effectively mitigate intestinal inflammation and promote wound healing by regulating gut microbiota homeostasis and modulating immune responses.^[^ ^10^ ^]^ Although many their active constituents have demonstrated promising therapeutic potential in experimental models of UC, their broader application remains constrained by limitations such as poor solubility, short half‐life, and low selectivity.

Dahuang Huanglian Xiexin Decoction is a well‐known classical prescription in Chinese medicine, renowned for its potent heat‐clearing and detoxifying effects. The combination of Coptis (Huanglian) and Rhubarb (Dahuang) is a common practice in Chinese medicine. Research has demonstrated that the principal active compound of Coptis, berberine (BBR), possesses a range of beneficial properties, including anti‐inflammatory,^[^ ^11^ ^]^ anti‐oxidation^[^ ^12^ ^],^ and metabolic disorder.^[^ ^13^ ^]^ BBR has been shown to exhibit anti‐inflammatory actions in the treatment of UC by restoring the integrity of the intestinal epithelial barrier.^[^ ^14^ ^]^ However, its clinical application is significantly limited by factors such as poor drug stability, low solubility, and restricted targeting capabilities. Thus, it is imperative to develop strategies aimed at enhancing the solubility, loading capacity, and retention time of BBR to improve its therapeutic efficacy. Notably, dendritic macromolecules have emerged as promising candidates for novel drug delivery technologies due to their intrinsic cavity structures, surface‐enriched active functional groups, and controllable physicochemical properties.^[^ ^15^ ^]^ Furthermore, precise and efficient drug delivery systems that modulate gut immune microbiota are crucial for successful clinical translation.^[^ ^16^ ^]^ As the primary active constituents of Rhubarb, Rhubarb polysaccharides (RP) possess adhesive properties, multifunctional groups, and intestinal targeting capabilities, which enhance their inherent characteristics and enable them to meet various applications. More importantly, studies have demonstrated that RP exhibits pharmacological activities such as gut microbiota regulation, and anti‐inflammatory and immune‐enhancing effects, making it an ideal candidate for the treatment of UC.^[^ ^17^ ^]^

**Scheme 1:**
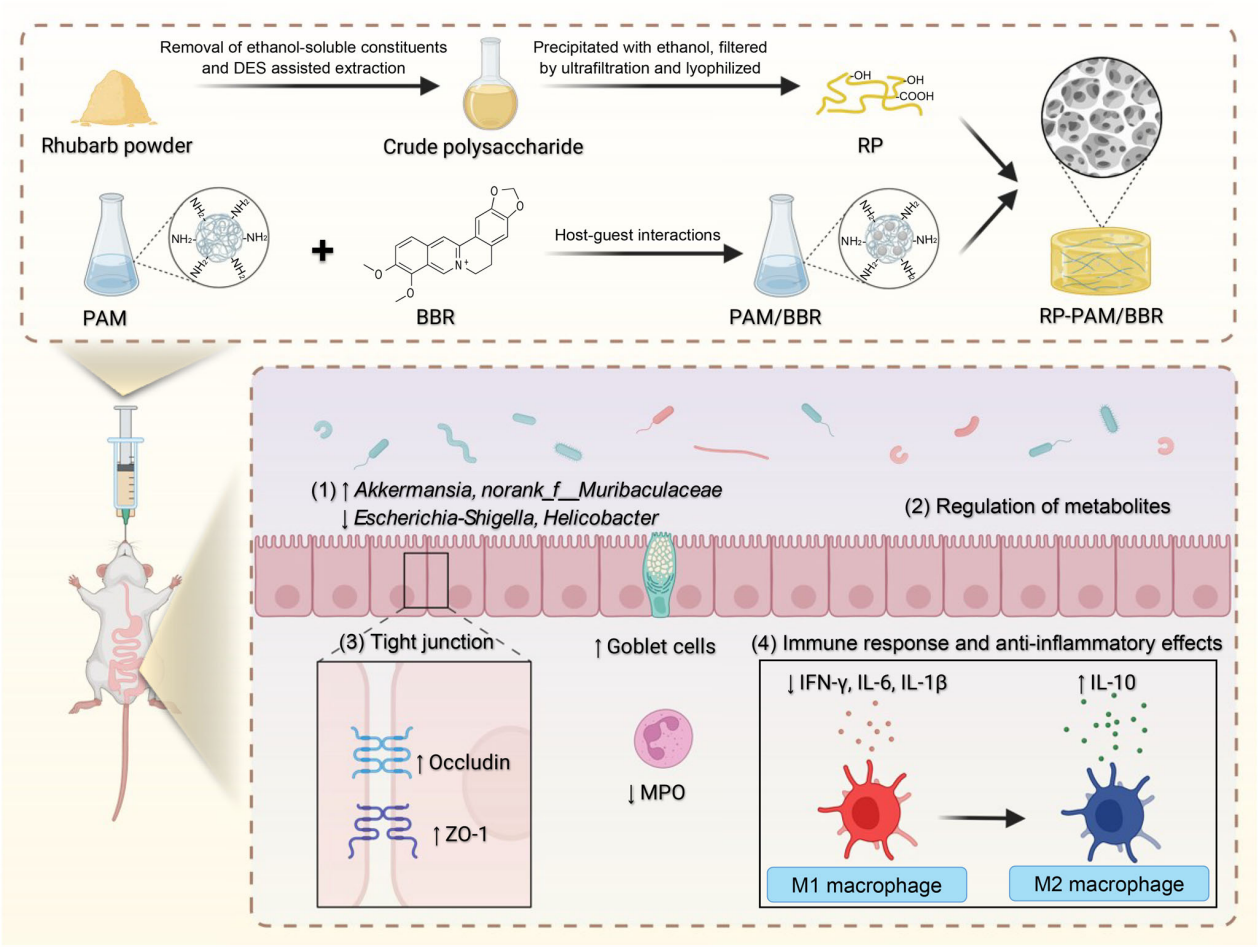
Schematic illustration of the preparation process for the polysaccharide‐berberine armored hydrogel (RP‐PAM/BBR) delivery system and its mechanism for mitigating colitis.

Building on the synergistic effects of key bioactive components in classic Chinese medicine prescription, we developed a porous drug delivery hydrogel system consisting of RP and BBR. This system is designed for oral administration to specifically target the gut, with the goal of modulating the gut microbiota and regulating the body's immune response (**Scheme** **1**). Specifically, a solution system with polyamidoamine dendrimer (PAMAM) encapsulating BBR was developed based on host‐guest interactions to enhance the water solubility of BBR. Subsequently, the system was combined with the extracted RP through hydrogen bonding and electrostatic interactions to construct a porous hydrogel. Following oral administration, the complex demonstrated significant long‐term retention effects. Given the critical roles of intestinal immune responses, gut microbiota composition, and microbial metabolites in the pathogenesis of UC, we further investigated the interplay among the host immune system, gut microbiota, and metabolome. Our research provides a safe and effective candidate for clinical UC treatment.

## 2 Results and Discussion

### 2.1 Design and Fabrication of the RP‐PAM/BBR Hydrogel

**Figure 1:**
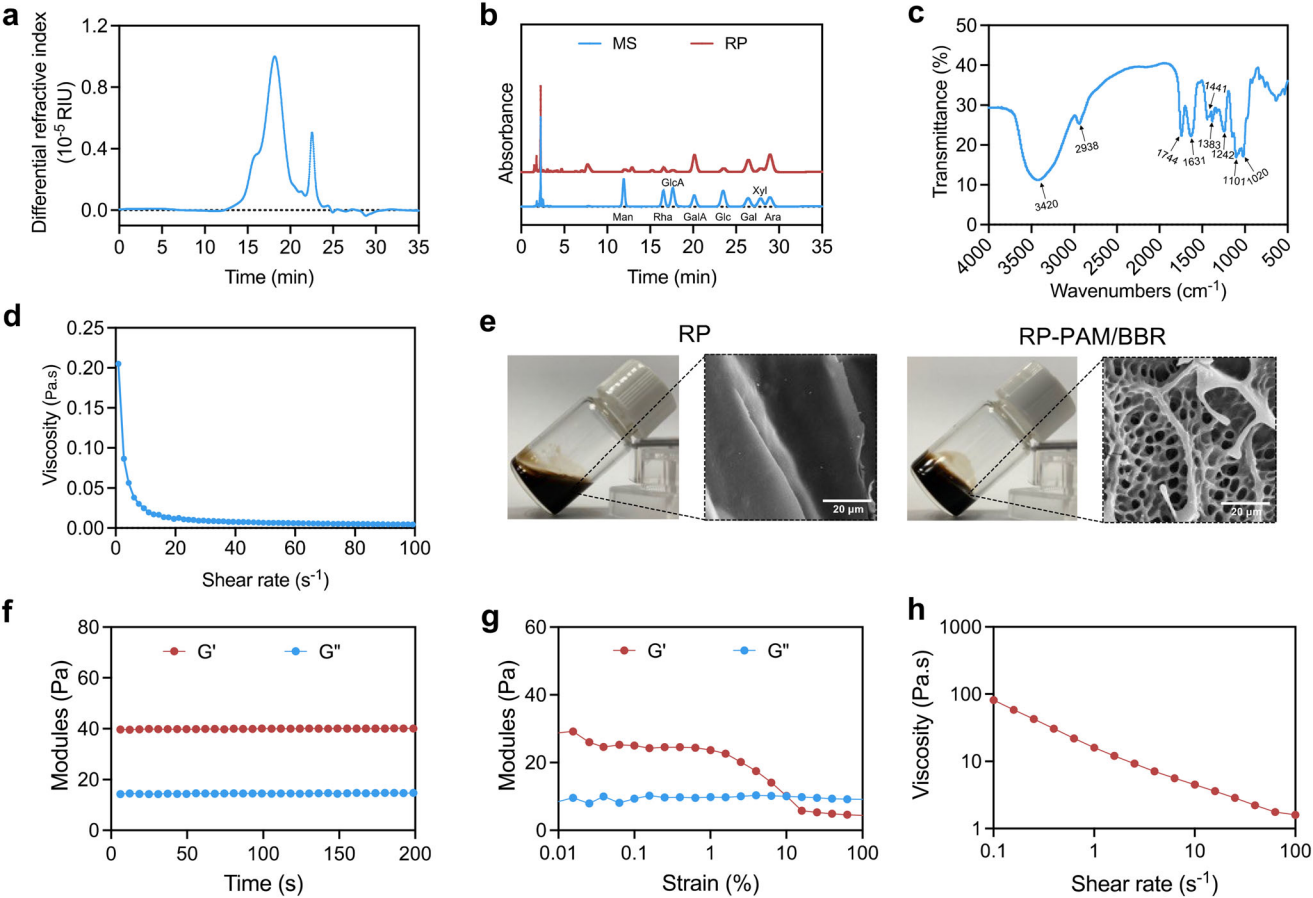
Characterization of the RP‐PAM/BBR hydrogel. a) HPSEC chromatograms of RP. b) HPLC chromatograms of RP. c) FTIR spectrum of RP. d) Apparent viscosities of RP. e) Photographs and SEM images of RP and RP‐PAM/BBR hydrogel. f) Time‐dependent rheology measurement of the RP‐PAM/BBR hydrogel. g) Strain‐dependent rheology measurement of the RP‐PAM/BBR hydrogel with the strain sweeping from 0.01% to 100% at an angular frequency of 10 rad s^−1^. h) Shear‐thinning property of the RP‐PAM/BBR hydrogel.

RP was extracted using a deep eutectic solvent (DES)‐assisted extraction method. Subsequently, PAMAM encapsulating BBR (PAM/BBR) was incorporated to fabricate an RP‐PAM/BBR hydrogel featuring a robust 3D network structure. Given that the chemical properties of polysaccharides play a critical role in their pharmacological characteristics, this study analyzed key parameters including molecular weight (*M_w_*), monosaccharide composition, Fourier transform infrared spectroscopy (FTIR) absorption profiles, viscosity, and morphological characteristics. As shown in **Figure** **1**a, high‐performance size exclusion chromatography (HPSEC) analysis showed that the *M_w_* of RP was 3.834 × 10^5^ Da. Compared with the acetylation results of the monosaccharide mixed standard (MS), the RP sample is mainly composed of arabinose, galactose, and galacturonic acid (Figure 1b). The FTIR spectrum (Figure 1c) of the RP presented the characteristic absorption bands at 3420 cm^−1^, which suggested that there are multiple hydroxyl groups and indicated the presence of intermolecular or intramolecular O─H bonds. The absorption bands at 2938 and 1441 cm^−1^ were the stretching vibration peak and bending vibration peak of methylene, respectively. The characteristic absorption bands at 1631cm^−1^ were assigned to the carbonyl group. The peak at 1383–1020 cm^−1^ indicated the presence of a different form of vibration in the C─O bond of the pyranose ring. The impact of shear rate on the apparent viscosities of RP is illustrated in Figure 1d, revealing a shear‐thinning fluid flow behavior as the shear rate increases from 0.1 to 100 s^−1^ due to the rearrangement and random coiling of RP molecules. The comprehensive physicochemical profiling establishes RP as a heteropolysaccharide with distinctive shear‐thinning properties and abundant hydroxyl/carbonyl functionalities. After incorporating BBR into PAMAM, the resulting solution (PAM/BBR) appears clearer than BBR in water (with undissolved powder at the bottom of the bottle) (Figure S1, Supporting Information), which is attributed to the unique structure of PAMAM that enhances the solubility of BBR in water through host‐guest interactions. Macroscopically, RP‐PAM/BBR exhibits a more stable state compared to RP. Scanning electron microscopy (SEM) reveals that RP possesses a layered architecture, while RP‐PAM/BBR features a porous scaffold structure (Figure 1e). This difference suggests that the amino functional groups of PAMAM and the hydroxyl groups on the surface of RP engage in hydrogen bonding and electrostatic interactions, contributing to the formation of a more stable porous structure. To elucidate the rheological characteristics of the RP‐PAM/BBR composite hydrogel, systematic analyses were conducted with a focus on viscoelastic behavior, thixotropic recovery capacity, and shear‐responsive viscosity modulation. Rheological assessments indicate that the storage modulus (G′) of RP‐PAM/BBR consistently exceeds the loss modulus (G″) over a duration of 200 seconds, demonstrating that the liquid phase of RP‐PAM/BBR exhibits gel‐like viscoelastic properties (Figure 1f). Furthermore, the thixotropic behavior of RP‐PAM/BBR leads to hydrogel collapse at 10% strain (Figure 1g). Figure 1h illustrates the relationship between shear rate and the apparent viscosities of RP‐PAM/BBR, revealing a shear‐thinning behavior as the shear rate increases from 0.1 to 100 s^−1^. Rheological and morphology analyses collectively demonstrate that the coordinated self‐assembly of RP polysaccharides with PAM‐mediated BBR facilitates the formation of hierarchically ordered networks, exhibiting superior mechanical stability. Moreover, the inclusion of polysaccharides optimizes the hydrogel for efficient oral delivery.

### 2.2 Biodistribution of the Hydrogel

**Figure 2:**
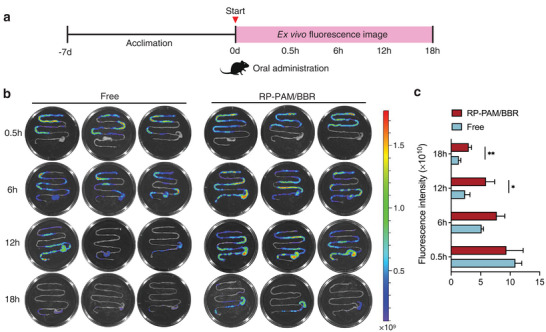
The retention and colon‐specific accumulation of RP‐PAM/BBR in vivo. a) The experimental design for in vivo imaging. b) The fluorescent pictures of mouse intestines in Free and RP‐PAM/BBR groups. c) The intensity analysis of fluorescence for mouse colons in each group following oral administration. Data are expressed as mean ± SD, with each experimental group consisting of three biological replicates (*n* = 3). *p < 0.05 and **p < 0.01.

To rigorously evaluate the colon‐targeting efficacy of the RP‐PAM/BBR hydrogel, DiR was incorporated into the hydrogel matrix as a fluorescent marker. The in vivo biodistribution of the hydrogel was subsequently monitored using a fluorescence imaging system. Mice were orally administered either free DiR or DiR‐RP‐PAM/BBR hydrogel to assess the hydrogel's targeted delivery to inflamed colonic sites. The gastrointestinal (GI) tract was harvested at 0.5, 6, 12, and 18 h post‐administration for ex vivo fluorescence imaging (**Figure** **2**a; Figure S2, Supporting Information). The RP‐PAM/BBR hydrogel was observed to transit rapidly through the stomach within 0.5 h, showing predominant localization in the ileum and cecum during the initial 6 h, and subsequently migrating toward colon (Figure 2b). Notably, at the 6‐h mark, the fluorescence intensity in the RP‐PAM/BBR group was higher compared to that in the control group. In vitro fluorescence imaging of the GI tract at this time point demonstrated that the RP‐PAM/BBR hydrogel effectively resisted degradation by digestive fluids, thereby facilitating the delivery of RP and BBR to the colon. Moreover, fluorescence signals remained detectable in the mice at 12 and 18 h post‐administration. The fluorescence observed in the GI tract of the RP‐PAM/BBR group was both more intense and of longer duration compared to that of the control group (Figure 2c), underscoring the hydrogel's enhanced inflammation‐targeting capability and prolonged retention time within the colon. In vitro release studies showed that RP‐PAM/BBR releases more slowly than PAM/BBR in both simulated gastric fluid (SGF) and simulated intestinal fluid (SIF) (Figure S3, Supporting Information). In SIF, the release reached equilibrium after 6 h and persisted for 18 h. These results indicate that RP‐PAM/BBR is relatively stable, with minimal release during gastric transit, enabling prolonged action upon reaching the colon. These findings further corroborate the RP‐PAM/BBR hydrogel's resistance to digestive fluids and its effective protection of the colon. This enhanced protective effect highlights the potential of polysaccharide‐based hydrogels as a reliable and efficient delivery system for targeted treatment of colonic disorders.

### 2.3 Therapeutic Efficacy of RP‐PAM/BBR in DSS‐Induced Acute Colitis

**Figure 3:**
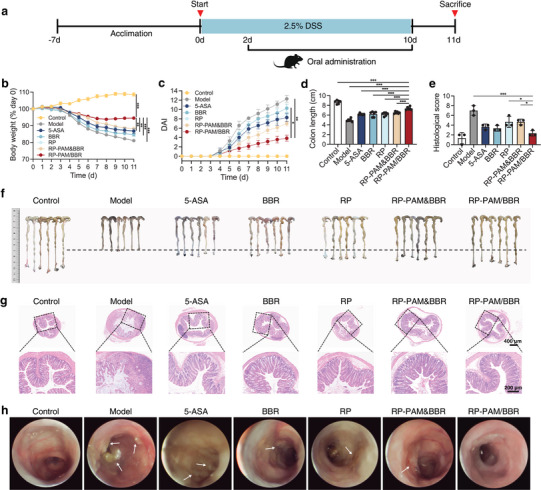
Therapeutic efficacy of RP‐PAM/BBR in DSS‐induced acute colitis. a) The experimental design for UC treatment. b) Variation of body weight during infection and treatment for 11‐day intervention. c) DAI trend over time. d) Average lengths of colons isolated from mice. e) Histological score. f) Photographs of the colon in different groups of mice. g) H&E staining of colon tissues from different groups, with low‐magnification (top) and high‐magnification (bottom) images. h) Endoscopic images of the colon in different groups of mice. Data are expressed as mean ± SD (*n* = 3 or 6). *p < 0.05, **p < 0.01, and ***p < 0.001.

In vivo, biodistribution results confirmed that the polysaccharide‐berberine armored hydrogel preferentially adheres to the colon and exhibits sustained‐release properties. Consequently, further investigations were undertaken to evaluate its potential as an enhanced therapy for acute colitis. For this purpose, dextran sulfate sodium (DSS)‐induced colitis mice were selected for subsequent in vivo experiments, as illustrated in **Figure** **3**a. Mice were administered 2.5% DSS for 10 consecutive days to induce acute colitis, followed by oral gavage of various interventions every other day from day 2 to day 10. Compared to the 5‐ASA, BBR, RP, and RP‐PAM&BBR groups, the RP‐PAM/BBR hydrogel effectively mitigated body weight loss in UC mice (Figure 3b) and reduced the disease activity index (DAI) (Figure 3c). In the model group, mice exhibited progressive weight loss at a rate of 18.91%, primarily due to colitis‐induced intestinal pathology that markedly reduced their food intake. This 15–20% weight loss range represents a hallmark pathophysiological response in acute UC modeling, consistent with prevailing research consensus.^[^ ^18^ ^]^ The RP‐PAM/BBR hydrogel effectively prevented the exacerbation of colon atrophy induced by DSS‐induced inflammation, as evidenced by a significantly longer average colon length in the RP‐PAM/BBR group compared to the model, 5‐ASA, BBR, RP, and RP‐PAM&BBR groups (Figure 3d,f). Histological analysis of hematoxylin and eosin (H&E)‐stained sections was conducted to evaluate the extent of inflammatory cell infiltration and epithelial cell damage. Fortunately, RP‐PAM/BBR treatment resulted in H&E staining that revealed no significant pathological changes (Figure 3e,g). The RP‐PAM/BBR group exhibited a significantly lower histological score compared to other groups, indicating that treatment with RP‐PAM/BBR effectively reverses DSS‐induced colon damage. Moreover, endoscopic imaging revealed that the colons in the UC model group displayed pronounced ulceration (Figure 3h). Spleen hypertrophy in DSS‐induced colitis mice was alleviated, as indicated by reduced spleen weight (Figure S4, Supporting Information). Following treatment, the 5‐ASA, BBR, RP, and RP‐PAM&BBR groups exhibited some improvement, but ulceration remained evident. In contrast, the colon images from the RP‐PAM/BBR group demonstrated a significant reduction in ulcer incidence, with colon morphology resembling that of healthy mice.

In the present study, we quantitatively assessed and compared the therapeutic efficacy of conventional 5‐ASA with a novel hydrogel‐based delivery system for the treatment of DSS‐induced colitis, employing a comprehensive set of colitis evaluation indices. The findings revealed that the RP‐PAM/BBR hydrogel demonstrated significantly enhanced therapeutic outcomes, notably by suppressing the shortening of colon length and alleviating tissue ulceration. Compared to the interventions using RP or BBR alone, the combined intervention better‐maintained colon length, controlled body weight, and improved DAI, showing the best outcomes in endoscopic imaging and histological scoring. Therefore, we further investigated the synergistic mechanisms of RP and BBR in the developed hydrogel system.

### 2.4 Restoration of Intestinal Barrier Function and Inhibition of Apoptosis

**Figure 4:**
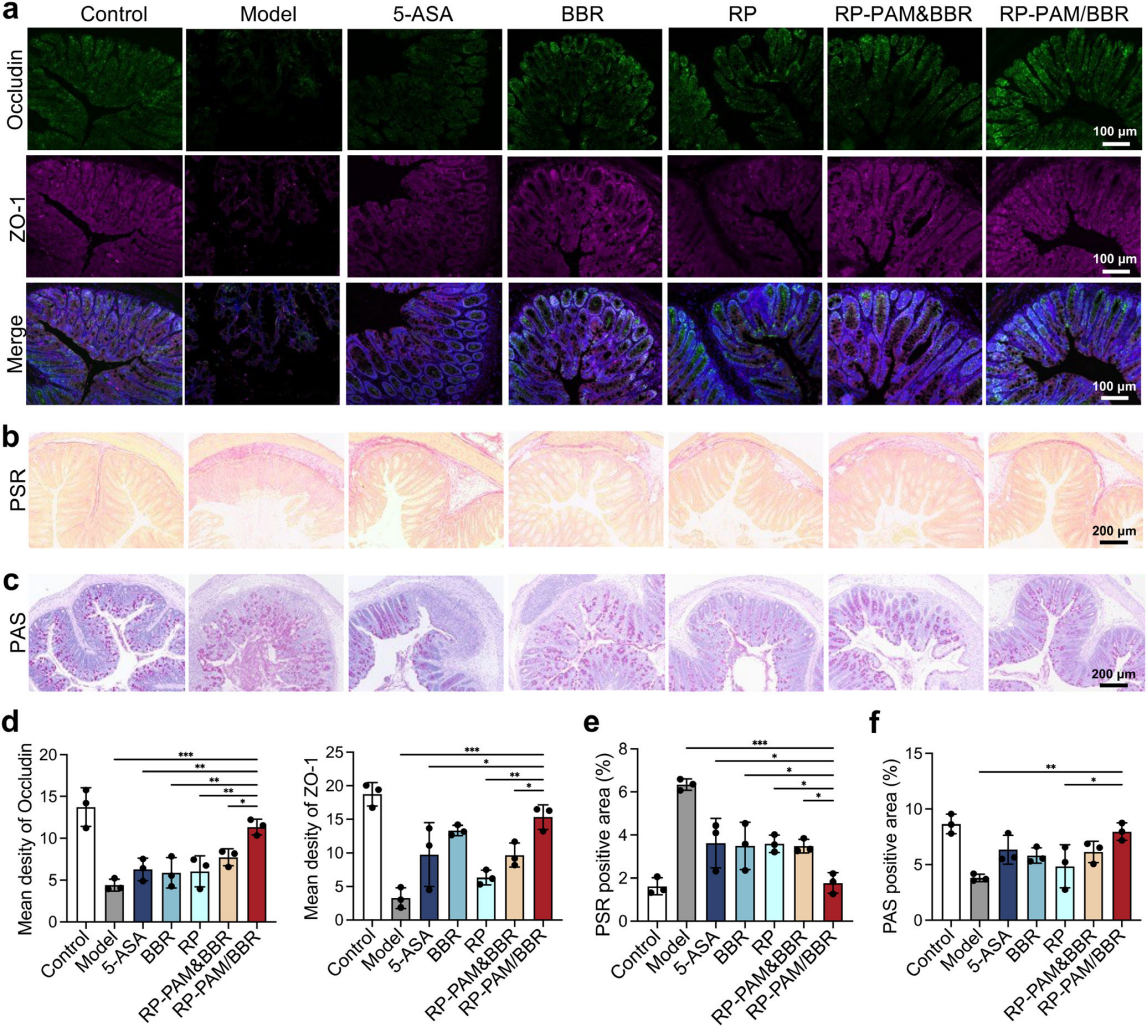
Restoration of intestinal barrier function and apoptosis inhibition. a) Typical immunofluorescence staining images of Occludin and ZO‐1 in colon tissues. b) The scanning results of PSR‐stained histological sections. c) The scanning results of PAS‐stained histological sections. d) The histogram of semi‐quantitative analysis of Occludin and ZO‐1 in colons. e) The histogram statistics of fibrosis area. f) The histogram statistics of the PAS‐positive area. Data are expressed as mean ± SD (*n* = 3). *p < 0.05, **p < 0.01, and ***p < 0.001.

The intestinal mechanical barrier is primarily comprised of intestinal mucosal epithelial cells and intercellular tight junctions.^[^ ^19^ ^]^ Tight junctions, in particular, serve as crucial connections between these epithelial cells and play a vital role in maintaining intestinal homeostasis.^[^ ^20^ ^]^ To assess the integrity of the epithelial barrier, we evaluated the colonic expression levels of tight junction proteins Occludin and ZO‐1. Immunofluorescence imaging (**Figure** **4**a,d) revealed significantly reduced fluorescence in the DSS model group, indicating a marked downregulation of Occludin and ZO‐1 expression in the colon. In contrast, the RP‐PAM/BBR‐treated group exhibited bright fluorescence for Occludin and ZO‐1, comparable to that observed in healthy mice. This finding suggests that RP‐PAM/BBR effectively promotes recovery of the intestinal epithelial barrier. At the same time, chronic inflammation in the colon often leads to intestinal fibrosis, a condition characterized by the excessive accumulation of extracellular matrix components that can result in strictures, reduced intestinal motility, and impaired nutrient absorption. Analysis of picrosirius red (PSR)‐stained sections and quantitative assessment showed that fibrosis was most pronounced in the DSS model group, with decreasing severity observed in the 5‐ASA, BBR, RP, and RP‐PAM&BBR groups (Figure 4b). Notably, the RP‐PAM/BBR‐treated mice demonstrated minimal fibrosis in their colon structure (Figure 4e). Furthermore, goblet cells, an essential type of intestinal cell, are distributed among the columnar epithelial cells of the mucosa. Their primary function is to synthesize and secrete mucins, which create a mucosal barrier to protect the epithelial cells.^[^ ^21^ ^]^ Periodic acid‐schiff (PAS) staining revealed extensive apoptosis of goblet cells in the colons of UC mice. While treatment with 5‐ASA, BBR, RP, and RP‐PAM&BBR all provided some degree of protection to goblet cells, RP‐PAM/BBR was the most effective in preserving goblet cell integrity (Figure 4c,f). These observations confirm the protective effect of RP‐PAM/BBR on the intestinal mucosa and further emphasize the importance of an intact intestinal barrier for protection against colitis.

### 2.5 Regulation of Immune Responses and Inflammation

**Figure 5:**
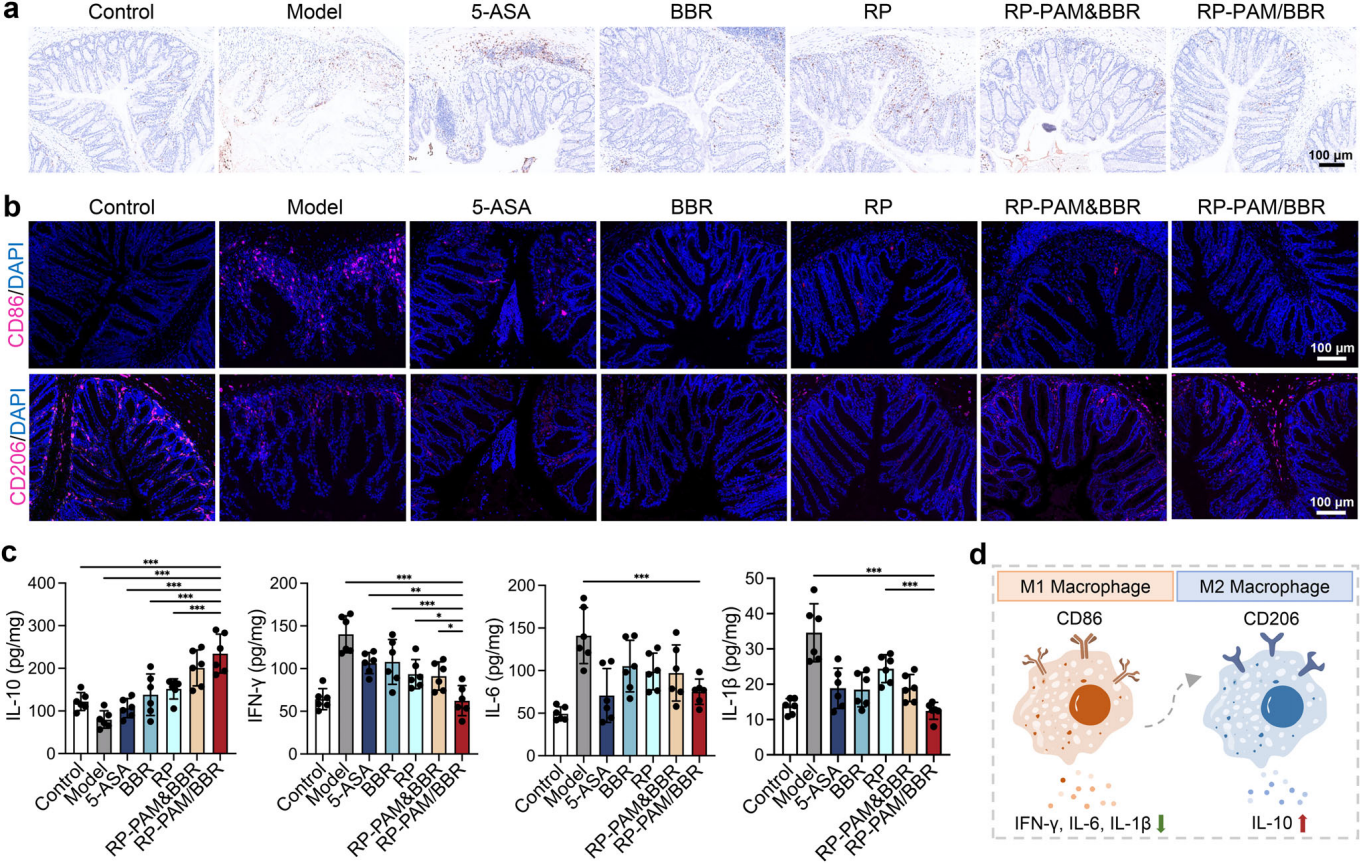
Regulation of immune responses and inflammation by RP‐PAM/BBR. a) Immunohistochemical staining of MPO in colon tissues. b) CD86 staining and CD206 staining of the colons. c) The levels of IL‐10, IFN‐*γ*, IL‐6, and IL‐1*β* in colon tissues isolated from different groups. d) Regulation of M2 macrophage polarization by RP‐PAM/BBR. Data are expressed as mean ± SD (*n* = 6). *p < 0.05, **p < 0.01, and ***p < 0.001.

Dysregulation of the immune response is a fundamental factor in the pathogenesis and progression of UC, contributing to an inappropriate and sustained inflammatory reaction that damages the intestinal mucosa.^[^ ^22^ ^]^ We then conducted an in‐depth analysis to figure out how RP‐PAM/BBR protects the mice from DSS‐induced colitis. Neutrophils, among the earliest infiltrating immune cells in UC, not only compromise the epithelial barrier through oxidative stress but also sustain inflammation by releasing cytokines and chemokines that enhance the proinflammatory response. Myeloperoxidase (MPO) is a prominent marker of neutrophil infiltration, with elevated MPO levels being closely associated with inflammation and tissue damage in colitis.^[^ ^23^ ^]^ Immunohistochemical (IHC) analysis demonstrated that treatment with RP‐PAM/BBR significantly reduced the MPO‐positive rate in colon tissues (**Figure** **5**a). This reduction in MPO positivity indicates that RP‐PAM/BBR effectively mitigates neutrophil‐driven inflammation in the colon, which supports the treatment's overall anti‐inflammatory effects. These findings are consistent with histological observations of reduced inflammatory cell infiltration, highlighting the therapeutic potential of RP‐PAM/BBR in ameliorating DSS‐induced colonic injury. To further elucidate the anti‐inflammatory mechanism of RP‐PAM/BBR, macrophage polarization in the colon was examined using immunofluorescence staining. The expression levels of M1 macrophages (CD86^+^) varied in the following order from highest to lowest: model, 5‐ASA, RP, RP‐PAM&BBR, BBR, and RP‐PAM/BBR. Conversely, M2 macrophages (CD206^+^) showed an opposing trend (Figure 5b). In the RP‐PAM/BBR‐treated group, there was a substantial presence of M2 macrophages (CD206^+^) within the colonic epithelium, indicating a shift toward an anti‐inflammatory and regenerative environment. The fluorescence intensity for M1 macrophages (CD86^+^) was nearly undetectable in the epithelial tissues, suggesting that RP‐PAM/BBR treatment effectively suppresses pro‐inflammatory macrophage activation. Additionally, the high presence of M2 macrophages may lead to an increased production of the anti‐inflammatory cytokine IL‐10 in the colon. To assess this, IL‐10 expression levels in colon tissues from different groups were measured using ELISA. The results indicated that RP‐PAM/BBR significantly elevated IL‐10 expression compared to other treatments (Figure 5c). Correspondingly, levels of inflammatory cytokines IFN‐*γ*, IL‐6, and IL‐1*β* were measured by ELISA. ELISA analysis revealed that in the DSS model group, expression levels of IFN‐*γ*, IL‐6, and IL‐1*β* were markedly upregulated. However, treatment with RP‐PAM/BBR normalized these cytokine levels to a profile similar to that of healthy controls, demonstrating its potent anti‐inflammatory capability. In summary, this polarization toward M2 macrophages likely contributes to the therapeutic efficacy of RP‐PAM/BBR in mitigating DSS‐induced colitis by reducing inflammation and promoting mucosal healing (Figure 5d).

### 2.6 Therapeutic Efficacy of RP‐PAM/BBR in DSS‐Induced Chronic Colitis Model

**Figure 6:**
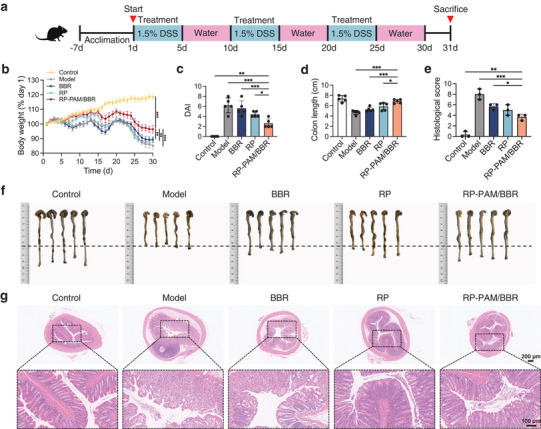
Therapeutic efficacy of RP‐PAM/BBR in DSS‐induced chronic colitis model. a) Schematic diagram illustrating the establishment of the DSS‐induced chronic colitis mouse model. b) Body weight variations in mice over the 30‐day treatment period. c) DAI score trends across groups. d) Colon length measurements for each experimental group. e) Pathological scores derived from H&E‐stained sections. f) Photographs of cecum‐colon tissues from each group. g) Histological images of H&E‐stained colon sections. Data are presented as means ± SD (*n* = 3 or 5). *p < 0.05, **p < 0.01, and ***p < 0.001.

To evaluate the long‐term therapeutic efficacy of RP‐PAM/BBR, we established a chronic colitis model in mice using three cycles of 5‐day 1.5% (w/v) DSS administration (5‐day exposure followed by 5‐day recovery intervals). Therapeutic interventions were administered via daily oral gavage during DSS challenge periods (**Figure** **6**a). While the model group exhibited characteristic disease manifestations including progressive body weight loss, elevated DAI scores, and significant colon shortening (Figure 6b–f), RP‐PAM/BBR treatment group displayed substantial attenuation of these pathological features. Histopathological analysis further indicated that RP‐PAM/BBR treatment effectively preserved colonic architecture, with mucosal inflammation scores comparable to healthy controls (Figure 6g). These collective findings demonstrate that RP‐PAM/BBR exerts robust protective effects against chronic colitis progression.

### 2.7 Regulation of the Intestinal Microbiota

**Figure 7:**
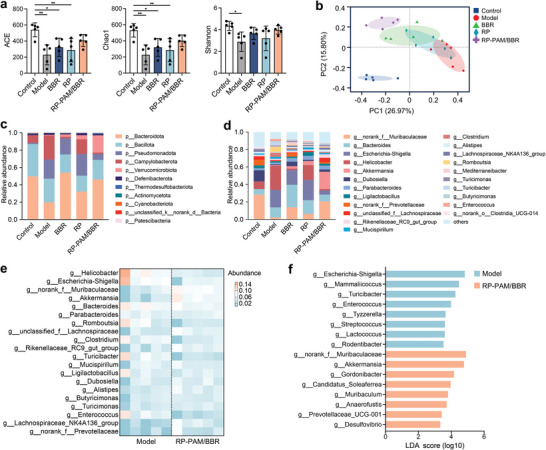
RP‐PAM/BBR partially recovers DSS‐mediated gut microbiome alteration. a) The *α*‐diversity index of ACE, Chao1 and Shannon. b) PCoA analysis at the Amplicon Sequence Variant (ASV) level. c) Phylum‐level taxonomic composition among groups. d) Genus‐level taxonomic composition among groups. e) Heatmap visualization of genus abundance patterns comparing model and RP‐PAM/BBR groups. f) LEfSe results at the genus level. Data are expressed as mean ± SD (*n* = 5). *p < 0.05, **p < 0.01, and ***p < 0.001.

In the context of IBD, dysbiosis characterized by an imbalance in gut microbial populations is closely linked to the onset and progression of inflammation. The depletion of beneficial bacteria and the overgrowth of pathogenic microorganisms can lead to an exacerbation of inflammatory responses. This dysregulation contributes to a breakdown of immune tolerance, resulting in chronic inflammation and tissue damage in the intestinal lining.^[^ ^3b^ ^]^ Therefore, the regulatory effects of RP‐PAM/BBR on gut microbiota were analyzed using 16S rRNA gene amplicon sequencing of fecal samples from mice with chronic colitis. The *α*‐diversity analysis revealed significant differences in the diversity and abundance of intestinal microorganisms between the model, BBR, and RP groups compared to the control group, as indicated by the ACE and Chao1 indices (**Figure** **7**a). In contrast, there were no significant differences in ACE, Chao1, and Shannon indices between the RP‐PAM/BBR group and the control group. This suggests that RP‐PAM/BBR positively influences the diversity of gut microbiota. Further investigation into the differences in gut microbial composition among the groups was conducted using Principal Coordinates Analysis (PCoA) to evaluate the overall state of the gastrointestinal microbiota (Figure 7b). The gut bacteria composition in the model group showed a clear shift in clustering compared to the control group, indicating that DSS administration disrupted gut microbiota homeostasis. Notably, the RP, BBR, and RP‐PAM/BBR groups progressively diverged from the model group, suggesting that RP‐PAM/BBR intervention tends to restore the gut microbiota structure altered by DSS. Subsequent analysis of gut microbiota structure at the phylum (Figure 7c; Figure S5, Supporting Information) and genus (Figure 7d; Figure S6, Supporting Information) levels revealed that the microbial composition between the control and RP‐PAM/BBR groups showed the highest similarities. Analysis of the relative abundance of microbiota at the phylum level exhibited that the relative abundance of *Bacteroidota* increased and that of *Pseudomonadota* and *Campylobacterota* decreased after RP‐PAM/BBR administration (Figure S7, Supporting Information). At the genus level, RP‐PAM/BBR administration increased the proportion of *norank_f__Muribaculaceae and Akkermansia* and effectively inhibited the proliferation of *Helicobacter* and *Escherichia‐Shigella* in the colon (Figure 7e; Figure S8, Supporting Information). Further Linear discriminant analysis Effect Size (LEfSe) analysis of genus levels revealed that RP‐PAM/BBR treatment indeed upregulated beneficial *Akkermansia* and *norank_f__Muribaculaceae* bacteria abundance (Figure 7f). Notably, *Akkermansia* functions as a critical symbiotic partner in gut homeostasis. Specifically, it stabilizes the intestinal immune microenvironment by suppressing hyperactivation of inflammatory cascades, including the TLR/NF‐κB and NLRP3 pathways.^[^ ^24^ ^]^ Concurrently, this commensal bacterium enhances epithelial barrier integrity through upregulation of tight junction proteins.^[^ ^25^ ^]^ Additionally, *norank_f_Muribaculaceae* mitigates inflammation, and suppresses harmful bacteria and oxidative stress, offering therapeutic potential for IBD management.^[^ ^26^ ^]^ Collectively, these findings demonstrate that RP‐PAM/BBR effectively modulates the gut microbiota by enhancing microbial diversity and specifically increasing beneficial taxa, particularly *Akkermansia*. This microbial remodeling contributes to the alleviation of intestinal inflammation in colitis.

### 2.8 Regulation Metabolites in DSS‐Induced Colitis Mice

**Figure 8:**
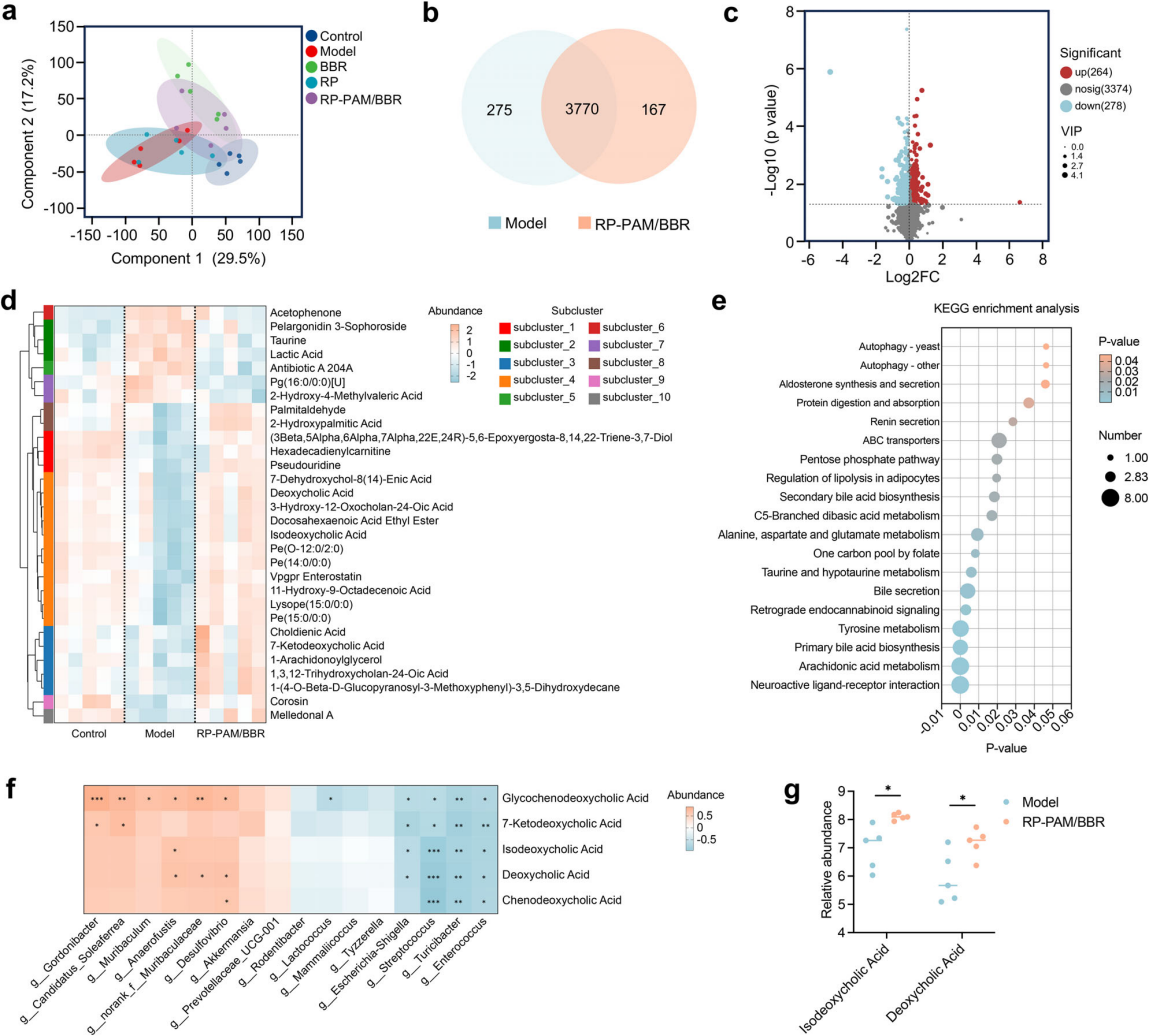
Fecal metabolomic alterations following RP‐PAM/BBR treatment. a) Clustering of metabolomics by PLS‐DA analysis. b) Venn diagram analysis comparing model and RP‐PAM/BBR groups. c) Volcano plot visualization of differentially abundant metabolites in the RP‐PAM/BBR group compared with the model group. d) Hierarchical clustering of the top 30 differential metabolites. e) KEGG enrichment scatter plot of RP‐PAM/BBR versus model groups based on differential metabolites. f) Correlation analysis among gut bacteria and metabolites. g) Comparative analysis of bile acid metabolites between RP‐PAM/BBR‐treated and model groups. Data are presented as means ± SD (*n* = 5). *p < 0.05, **p < 0.01, and ***p < 0.001.

Emerging evidence highlights that microbiota‐driven metabolic reprogramming plays a pivotal role in modulating intestinal immunity and mucosal homeostasis, with UC‐associated dysbiosis profoundly altering immunoregulatory metabolites such as short‐chain fatty acids^[^ ^27^ ^]^ and bile acids.^[^ ^28^ ^]^ To elucidate how RP‐PAM/BBR restores metabolic balance while alleviating colitis, untargeted metabolomics was performed in DSS‐challenged mice. Partial least squares‐discriminant analysis (PLS‐DA) revealed distinct clustering between healthy and colitis groups, with RP‐PAM/BBR treatment shifting the metabolic profile toward normalcy (**Figure** **8**a). To compare metabolite profiles, a Venn diagram was generated to illustrate the shared and distinct metabolites between the model and RP‐PAM/BBR groups (Figure 8b). Subsequent analysis revealed significant differences in metabolite composition. As depicted in the volcano plots (Figure 8c), the RP‐PAM/BBR group exhibited notable alterations in metabolite levels compared to the model group, with 264 metabolites significantly upregulated and 278 metabolites downregulated following RP‐PAM/BBR treatment. Clustering analysis of differentially expressed metabolites revealed that RP‐PAM/BBR treatment tends to restore the metabolite profile disrupted by DSS (Figure 8d). The top 30 differential metabolites were predominantly grouped in clusters 3 and 4, with several metabolites associated with bile acid metabolism, including deoxycholic acid, isodeoxycholic acid, choldienic acid, and 7‐ketodeoxycholic acid. To elucidate the functional implications of the altered metabolites, the Kyoto Encyclopedia of Genes and Genomes (KEGG) pathway enrichment analysis was conducted (Figure 8e). The results revealed enhanced primary and secondary bile acid biosynthesis, while pro‐inflammatory pathways, including arachidonic acid and tyrosine metabolism, were significantly downregulated. Crucially, Spearman correlation linked elevated secondary bile acids, including isodeoxycholic acid and deoxycholic acid to enriched *Anaerofustis* and *norank_f__Muribaculaceae*, while inversely correlating with pathogenic *Escherichia‐Shigella* and *Streptococcus* (Figure 8f). Mechanistically, RP‐PAM/BBR‐mediated deoxycholic acid elevation likely suppresses *Escherichia‐Shigella* overgrowth,^[^ ^29^ ^]^ while isodeoxycholic acid promotes colonic Treg differentiation to resolve chronic inflammation.^[^ ^30^ ^]^ The expression differences of isodeoxycholic acid and deoxycholic acid between the two groups are illustrated in Figure 8g. These findings establish that RP‐PAM/BBR coordinates microbiota‐metabolite crosstalk to reinstate immunometabolic homeostasis in colitis.

### 2.9 Safety of Oral RP‐PAM/BBR Hydrogel

**Figure 9:**
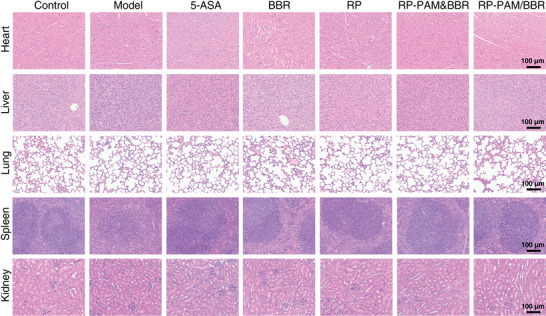
The scanning images of H&E staining of major internal organs, including the heart, liver, lungs, spleen, and kidneys of mice.

Considering the potential side effects, the safety of the polysaccharide‐berberine armored gel was evaluated. At the end of the experiment, mice were euthanized, and major organs, including the heart, liver, lungs, spleen, and kidneys, were excised for histopathological analysis using H&E staining to evaluate in vivo toxicity. As illustrated in **Figure** **9**, oral administration of the RP‐PAM/BBR hydrogel did not induce significant pathological alterations or inflammatory lesions in any of the examined organs compared to the healthy control group. These findings suggest that the RP‐PAM/BBR hydrogel possesses sufficient biocompatibility. Consequently, it holds potential as an oral therapeutic agent for investigating the preventive effects of RP and BBR in protecting against the development of colitis.

## 3 Conclusion

UC is a multifactorial, polymicrobial disease driven by complex interactions between host immune responses, gut microbiota dysbiosis, and compromised intestinal barrier integrity.^[^ ^31^ ^]^ Emerging evidence suggests that combination therapies, which leverage synergistic interactions among therapeutic components, offer superior efficacy for complex conditions such as chronic inflammation, metabolic disorders, and neurodegenerative diseases compared to monotherapies. The interactions between these various therapeutic components can modify key properties, such as size, morphology, and charge, leading to synergistic effects and promoting targeted delivery. In the present study, PAMAM and BBR interact through host‐guest interactions between the hydrophobic cavity and hydrophobic molecules, significantly enhancing the solubility of BBR. The resulting PAM/BBR complex then forms a polysaccharide‐berberine armored hydrogel with RP via hydrogen bonding and electrostatic interactions, aimed at modulating intestinal inflammation, altering gut microbiota composition, and restoring metabolite profiles. This polysaccharide‐rich hydrogel, specifically designed for colon‐targeted protection, exhibits pronounced mucoadhesive properties that facilitate targeted accumulation on the colonic mucosa. Our findings demonstrate that RP‐PAM/BBR significantly alleviates intestinal inflammation, as evidenced by a 66.7% reduction in inflammation scores based on histological analysis and a marked decrease in pro‐inflammatory cytokine levels. Additionally, the hydrogel promotes immune homeostasis by enhancing M2 macrophage polarization. In terms of microbiota regulation, 16S rRNA sequencing revealed that RP‐PAM/BBR restores microbial diversity, increasing the abundance of beneficial taxa such as *Akkermansia* and *norank_f__Muribaculaceae* while reducing pathogenic populations. Metabolomics profiling further confirmed the hydrogel's role in normalizing bile acid metabolism, with significant upregulation of deoxycholic acid and isodeoxycholic acid, which likely contributes to its anti‐inflammatory effects. Table S1 (Supporting Information) provides a systematic comparison between our findings and prior related studies, highlighting the superior therapeutic efficacy and comprehensive mechanistic exploration achieved in our work. Future investigations will focus on optimizing the hydrogel's viscoelastic properties and degradation profiles to enable personalized therapeutic regimens. Furthermore, exploring the therapeutic potential of hydrogel in other inflammatory conditions, such as rheumatoid arthritis or metabolic syndrome, could broaden its translational impact and contribute to the development of versatile, microbiota‐targeted therapies.

## 4 Experimental Section

### Material and Reagents

BBR was purchased from Shanghai Yuanye Biological Technology Co., Ltd. (Shanghai, China). 5‐ASA was obtained from Macklin (Shanghai, China). Primary amine surface PAMAM dendrimers (4th generation) were provided by Dendritech, Inc. (Midland, MI, USA). DiR Iodide was supplied by Bridgen Biotechnology Co., Ltd. (Beijing, China). ELISA kits for mice IL‐10, IFN‐*γ*, IL‐6, and IL‐1*β* were manufactured by BioLegend, Inc. (San Diego, CA, USA). The antibodies for immunofluorescence were obtained from Proteintech Group, Inc. (Wuhan, China). All other reagents were analytical grade without further purification.

### Extraction and Characterization of the RP

Rhubarb polysaccharides were extracted by using DES‐assisted extraction based on the previously established methodologies.^[^ ^32^ ^]^ DES was prepared using choline chloride and ethylene glycol in a molar ratio of 1:3. The extraction procedure is as follows: initially, 5.0 g of powdered sample is subjected to reflux with ethanol (50.0 mL, 80%, v/v) for 1 h. Subsequently, 90.0 mL of deionized water and 60.0 mL of the deep eutectic solvent are added to facilitate a 2‐h extraction process. Following centrifugation, polysaccharides from the extract are separated utilizing graded alcohol precipitation and membrane filtration techniques. Finally, impurities are removed via ultrafiltration in deionized water, with a molecular weight cutoff of 3.0 kDa.

The *M_w_* of RP and its isolated fractions were measured by high‐performance size exclusion chromatography coupled with multi‐angle laser light scattering and refractive index detector. The monosaccharide composition of RP was analyzed using an Ultimate U3000 LC system (Thermo Fisher Scientific, Waltham, MA, USA) equipped with a Phenomenex Gemini C18 column (150 mm × 4.6 mm, 5 µm). The mobile phase consisted of two components: phase A was 17% acetonitrile, while phase B comprised 83% potassium dihydrogen phosphate (0.05 m; pH 6.70) in water. The flow rate was maintained at 1 mL·min^−1^, and detection was performed at a wavelength of 245 nm. The FTIR of the RP was recorded in the range of 4000–400 cm^−1^ using a Nicolet iS10 FTIR spectrometer (Thermo Fisher Scientific, Waltham, MA, USA). The RP morphology was characterized by SEM (FEI Sirion 200, Thermo Fisher Scientific, Waltham, MA, USA).

### Preparation and Characterization of RP‐PAM/BBR Hydrogel

Primary amine surface PAMAM dendrimers were mixed with deionized water at a volume ratio of 8:2 and used as the stock solution. The addition of BBR to the PAMAM solution formed the PAM&BBR complex through non‐sonication and the stable PAM/BBR complex via 2 h of sonication, respectively. 400 mg of RP was dissolved in 3 mL of deionized water, followed by the addition of 1 mL of the PAM/BBR complex, and the mixture was stirred thoroughly to obtain the RP‐PAM/BBR hydrogel.

A camera was used to record the macroscopic appearance of RP and RP‐PAM/BBR hydrogel. The RP‐PAM/BBR hydrogel was freeze‐dried, sprayed with gold, and then characterized by SEM. The hydrogels were confirmed by FTIR spectrophotometer. Rheological properties were investigated using a Discovery Hybrid Rheometer‐2 (TA Instruments, New Castle, DE, USA). First, time‐dependent rheology measurement of the RP‐PAM/BBR was conducted. For strain‐dependent rheology, the strain swept from 0.01% to 100% at an angular frequency of 10 rad·s^−1^. A continuous step strain measurement of the RP‐PAM/BBR hydrogel was carried out to assess its thixotropic properties. Furthermore, the viscosity of the RP‐PAM/BBR hydrogel was measured as a function of shear rate 0.1 to 100 s^−1^.

### In Vitro Drug Release

The release profile of BBR from the RP‐PAM/BBR hydrogel was assessed using a dialysis setup in SGF (pH 1.2) and SIF (pH 6.8). Briefly, 1 mL of free BBR or RP‐PAM/BBR hydrogel was sealed in dialysis membranes (3.5 kDa cutoff) and immersed in 15 mL of SGF or SIF at 37 °C with agitation at 120 rpm. At set intervals (1, 2, 4, 6, 12, 18, and 24 h), 0.4 mL of medium was sampled, replaced with fresh medium, and analyzed for BBR content using UPLC (Waters Corp., Milford, MA, USA) with PDA detection at 345 nm. Cumulative release percentages were calculated and expressed as mean ± SD (*n* = 3).

### Mouse Experiments

All animal experiments were conducted in strict accordance with the ethical guidelines and regulations approved by the Committee on the Use and Care of Animals at Zunyi Medical University (Ethics Approval No.: ZHSC‐2‐2023017). Male C57BL/6 mice, aged 7 weeks, were procured from the Guangdong Zhiyuan Biomedical Technology Co., Ltd. (Guangzhou, China) in this study. The mice were acclimatized for one week in a temperature‐controlled environment (22–25 °C) with a 12‐h light‐dark cycle, and provided with adequate food and water for maintenance. The maintenance diet was supplied by Beijing HFK Bio‐Technology Co., Ltd. (Beijing, China). Throughout the experiment, mice were monitored daily and humanely euthanized at the study endpoint using CO_2_ asphyxiation.

### Mouse Experiments: In Vivo Distribution Studies

For the in vivo distribution and retention study, DiR (50 µg·mL^−1^) was loaded in the delivery system. Male mice were randomly divided into two groups: Free DiR and DiR‐RP‐PAM/BBR groups (*n* = 3 per group), with three independent biological replicates. Then, Free DiR and DiR‐RP‐PAM/BBR were orally administered to the mice in the two groups, respectively. Mice were euthanized and gastrointestinal tissues were collected at four predetermined time points (0.5, 6, 12, and 18 h) after oral administration. Gastrointestinal tissues from different groups were scanned using an in vivo imaging system (Lumina XR III, Horiba Scientific, Edison, NJ, USA).

### Mouse Experiments:Therapeutic Effects of RP‐PAM/BBR in DSS‐Induced Acute Colitis In Mice

The male C57BL/6 mice were stochastically formed into seven groups (*n* = 6 per group), including control, model, 5‐ASA, BBR, RP, RP‐PAM&BBR, and RP‐PAM/BBR groups. Next, the mice in control group were given normal deionized water. The experimental animals in the model, 5‐ASA, BBR, RP, RP‐PAM&BBR, and RP‐PAM/BBR groups were ensured to drink DSS aqueous solution (2.5%, w/v) for 10 days without interruption to establish experimental colitis model. From days 2 to 10, the animals in control, 5‐ASA, BBR, RP, RP‐PAM&BBR, and RP‐PAM/BBR groups were orally administered with phosphate‐buffered saline (PBS), 5‐ASA (100 mg·kg^−1^), BBR, RP, RP‐PAM&BBR, and RP‐PAM/BBR (the dosage calculated as RP concentration was 100 mg·kg^−1^) once every other day, separately. The DAI and weight changes of experimental individuals in each group were recorded every day. Assessment of DAI included weight loss (0 to 4), stool consistency (0 to 4), and stool bleeding (0 to 4). At the end of the experimental period, the pathological state of the ulcer in the colon of the mice was observed through endoscopy. The colon tissues and main organs (heart, liver, spleen, lungs, and kidneys) were picked.

### Mouse Experiments:Therapeutic Effect of RP‐PAM/BBR on DSS‐Induced Chronic Colitis in Mice

A chronic colitis model was induced in C57BL/6 mice through three cyclic regimens of 1.5% (w/v) DSS administered ad libitum for 5 consecutive days, each followed by a 5‐day recovery period with normal water access. During DSS exposure phases, therapeutic interventions (BBR, RP, RP‐PAM/BBR hydrogel or controls) were delivered via daily oral gavage (*n* = 5 per group).

### Histopathology Studies

Tissue samples were fixed in 4% paraformaldehyde, embedded in paraffin, and sectioned into 4 µm slices for H&E, PSR, and PAS staining. The stained sections were observed using a fluorescence microscope to assess the damage levels in the colon. The histopathological analysis includes the assessment of inflammation severity, depth of tissue damage, extent of crypt injury, and percentage of affected tissue.

### Immunofluorescence Evaluation

Immunofluorescence staining was performed to analyze the expression of Occludin, ZO‐1, CD86, and CD206 in the distal colon segments. The sections were incubated with specific primary antibodies targeting Occludin, ZO‐1, CD86, and CD206. Subsequently, HRP‐linked secondary antibodies were applied to facilitate detection. To enhance signal amplification, the sections were stained with Cy3‐Tyramide. The cell nuclei were counterstained with DAPI to provide contrast. Finally, the stained sections were examined using a Nikon Eclipse C1 laser scanning confocal microscope (Nikon Instruments Inc., Tokyo, Japan), which allowed for the visualization and quantification of the expression levels of these markers.

### Immunohistochemistry Analysis

To assess the expression of MPO in the intestines, IHC analysis was performed according to the previously established protocol. Visualization of the staining was achieved using diaminobenzidine, followed by counterstaining with hematoxylin. The stained sections were examined under a Nikon Eclipse E100 microscope.

### Measurement of Inflammatory Cytokines in Colon Tissue

To measure inflammatory cytokines in colon tissue, harvested tissues were homogenized in ice‐cold PBS, and the homogenates were centrifuged to obtain tissue lysates. Cytokines including IL‐10, IFN‐*γ*, IL‐6, and IL‐1*β* were quantified using ELISA kits according to the manufacturer's protocol.

### 16S rRNA Gene Sequencing and Data Analysis

Fecal samples were collected and stored at −80 °C until DNA extraction. The V3‐V4 hypervariable regions of the 16S rRNA gene were amplified by PCR with primers 341F and 806R. The amplified products were quantified using the QuantiFluor™‐ST Fluorescence Quantitation System (Promega Corporation, Madison, WI, USA), and equimolar amounts of the purified amplicons were pooled for sequencing on the Illumina MiSeq platform. Sequence data were processed through the QIIME2 pipeline, which included adapter trimming with Cutadapt, quality filtering, and denoising with DADA2 to identify ASVs. Taxonomic classification was conducted using the SILVA database. Statistical analyses and visualizations, such as *α*‐diversity, *β*‐diversity, and PCoA, were conducted by Majorbio Bio‐Pharm Technology Co., Ltd. (Shanghai, China) to interpret microbial community structures.

### Metabolomics Analysis

Fecal samples were subjected to untargeted metabolomics profiling via liquid chromatography‐mass spectrometry. To characterize metabolic alterations following RP‐PAM/BBR treatment, a suite of analytical approaches was employed, including PLS‐DA, Venn diagrams, volcano plots, KEGG pathway enrichment, microbiota‐metabolite correlation analyses, and identified significant differential metabolites.

### Statistical Analysis

GraphPad Prism 9.0 was used for statistical analysis, with all assay data presented as mean ± SD. Statistical significance was determined using one‐way analysis of variance (ANOVA) followed by Tukey's post hoc test for multiple comparisons. A *p*‐value of <0.05 was considered statistically significant.

## Conflict of Interest

The authors declare no conflict of interest.

## Supporting information



Supporting Information

## Data Availability

The data that support the findings of this study are available from the corresponding author upon reasonable request.
