# Supplementary material for: Colon‐Targeted Natural Polysaccharide‐Berberine Armored Hydrogel for the Treatment of Colitis
Source: Adv Healthc Mater. 2025 Jun 25;14(23):2404908. doi: 10.1002/adhm.202404908 (PMC12417751; doi:10.1002/adhm.202404908)
Supplement: Supplementary file 1 — Supporting Information [file ADHM-14-0-s001.docx]

**Supplementary Information for**

**Colon-Targeted Natural Polysaccharide-Berberine Armored Hydrogel for the Treatment of Colitis**

*Miao Guo, Bo Li, Hongyi Li, Yi Chen, Qin Yuan, Mingju Shui, Hefeng Zhou, Wei Hao*, Shengpeng Wang**


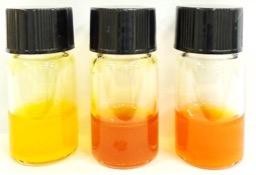


**Figure S1.** From left to right: BBR in water, PAM&BBR (passive mixing method) and PAM/BBR (ultrasonication-mediated method).

**Figure S2.** Fluorescent images of intestinal tissues in the Free and RP-PAM/BBR treatment groups (n = 6).

**Figure S3.** (a) Cumulative release of BBR in SGF (pH 1.2) (n = 3). (b) Cumulative release of BBR in SIF (pH 6.8) (n = 3).

**Figure S4.** (a) Photographs of representative spleens in different groups of mice. (b) Average weights of spleens isolated from mice (n = 6).

**Figure S5.** Histograms of phylum abundance of samples from each group (n = 5).

**Figure S6.** Histograms of genus abundance of samples from each group (n = 5).

**Figure S7.** Relative abundance of the individual phylum (n = 5).

**Figure S8.** Relative abundance of the individual genus (n = 5).

**Table S1.** Comparison of various targeted therapies for colitis

| Composition | Drug Delivery Mechanism | Therapeutic Outcomes | Microbiota and Metabolite Regulation | Immunomodulation | Study |
| --- | --- | --- | --- | --- | --- |
| Ascorbyl palmitate (AP) microfibers | Enzyme-triggered drug release; negatively charged fibers adhere to inflamed mucosa | Significant reduction in colitis scores | Not reported | Reduced pro-inflammatory cytokines; no specific immune cell data | Zhang et al. (2015, IT-Hydrogel) |
| CD–Cur–CANPs | Enzyme-triggered controlled release; colon-targeted accumulation for 12h | Mild relief of intestinal inflammation | Increased Bifidobacteria and Lactobacilli | Reduced pro-inflammatory cytokines; no specific immune cell data | Li et al. (2021, CD–Cur–CANPs) |
| Rhubarb polysaccharide and berberine co-assembled nanoparticles | Colon-targeted accumulation for 8h | Mild relief of intestinal inflammation | Increased Lactobacilli | Reduced pro-inflammatory cytokines; no specific immune cell data | Feng et al. (2023, BBR–DHP NPs) |
| Rhubarb polysaccharide + berberine-loaded PAMAM dendrimer | Self-assembled via hydrogen bonds and electrostatic interactions; mucoadhesive, colon-targeted accumulation for 18h | 36% reduction in histological score; restored colon length; alleviated mucosal ulceration | Increased Akkermansia; restored bile acid metabolism (e.g., deoxycholic acid upregulation) | Reduced neutrophil infiltration; Increase M2 macrophage; Suppressed IFN-γ, IL-6, and IL-1β | This study |
